# Weighing in on the Off-Label Use: Initial Experience of Neuroform EZ Stenting for Intracranial Arterial Stenosis in 45 Patients

**DOI:** 10.3389/fneur.2018.00852

**Published:** 2018-10-10

**Authors:** Zhihua Du, Jing Mang, Shengyuan Yu, Chenglin Tian, Xiangyu Cao, Xinfeng Liu, Renzheng Ma, Rongju Zhang, Bin Lv, Jun Wang

**Affiliations:** ^1^Department of Neurology, Chinese PLA General Hospital, Beijing, China; ^2^Department of Neurology, China-Japan Union Hospital of Jilin University, Changchun, China

**Keywords:** intracranial arterial stenosis, endovascular treatment, stent, Neuroform EZ, efficacy

## Abstract

**Background:** The role of stenting for intracranial arterial stenosis (ICAS) has been increasingly debated due to negative results of randomized trials. Thus, exploration of more appropriate devices may hopefully shed light on the endovascular approach, especially for patients with recalcitrant ICAS related to a high risk of stroke. We sought to present and analyze the data of Neuroform EZ stenting for medically refractory ICAS in a single-center series.

**Materials and methods:** Between November 2016 and January 2018, 45 consecutive patients treated with the Neuroform EZ stent were included in our retrospective study. Outcomes evaluation included successful procedure rate, vascular event within 30 days and recurrent stenosis for at least 6 months after the procedure.

**Results:** The technical success rate was 100% for all 46 stenotic lesions. Mean pre-stent stenosis was 86.5 ± 8.7%, improving to 23.7 ± 18.1% after stenting. Combined procedure related vascular event rate was 2.2% (*n* = 1) within 30 days after the procedure. No in-stent restenosis was observed during an average follow-up period of 7.3 months.

**Conclusion:** The Neuroform EZ stent system could serve as an off-label but promising optional device for ICAS stenting in a carefully selected subgroup of patients. Further longer-term clinical follow-up is mandatory to validate our initial results.

## Introduction

Intracranial arterial stenosis (ICAS) attributable to atherosclerosis is one of the most common causes of stroke worldwide (1). Therapeutic strategies for this high-risk disease include intensive management of risk factors, combination antiplatelet treatment, and endovascular therapy (1). As an optional treatment for symptomatic ICAS, elective percutaneous transluminal angioplasty and stenting (PTAS) had been introduced with the advent of the Gateway balloon/Wingspan stent system (2, 3). However, subsequent data from the randomized trial (SAMMPRIS) indicated that aggressive medical management was superior to PTAS with the use of the Wingspan stent system, because of the poor outcomes and high rates of perioperative complications of PTAS (4). Nevertheless, there are still a considerable number of patients with ICAS who remain at high risk of stroke in the real-world despite aggressive medical therapy (5–7). Taking the complications associated with device selection into careful consideration, the exploration for more safe and effective endovascular procedure with the new option of devices for this subgroup of patients challenges both neurologists and neurointerventionists (8–10). Recently, the Neuroform stent had been used for ICAS in several case series (11–13). Thus, for the relatively small sample size, beyond the scope of the indicated uses outlined in the device manual, the experience of the Neuroform EZ stenting for ICAS was still limited. The purpose of the present study was to respectively evaluate the feasibility and safety of this alternative procedure, and preliminarily provide the indications of the Neuroform EZ stent use for medically refractory ICAS in a single-center series.

## Patients and methods

### Patients

We retrospectively reviewed our patient database to identify individuals with ICAS which had been treated using the Neuroform EZ stent. Clinical and procedural data of included patients were examined by a stroke neurologist (RM) and a neurointerventionist (RZ) independently. The following data were recorded: demographic data, clinical presentation, lesion characteristics, procedural feasibility, complications and follow-up angiographic results. Before data extraction, the inclusion, and exclusion criteria had been defined as follows. Inclusion criteria: (1) Symptomatic ICAS (70–99% stenosis) with hypoperfusion of the stenotic arterial territory. (2) Aggressive medical management (dual antiplatelet medication for at least 3 months) failed to prevent recurrent low-flow TIAs, nondisabling ischemic stroke, or progressive stenosis. (3) The stenotic arterial territory had no sufficient collaterals. Exclusion criteria: (1) Non-atherosclerotic intracranial arterial stenosis, e.g., identified or suspected vasculitis or vessel dissection. (2) Acute cerebral infarction within two weeks. (3) Patients with a baseline modified Rankin score (mRS) of >3 points before the procedure. The demographic characteristics of patients were obtained from the hospital records, including age, gender and ethnicity. Lesion morphology was described as location, Mori classification and whether there was perforator involvement.

### Procedure

Angiography and interventional procedures were performed in the interventional suite with a biplane angiography system (Allura Xper FD20/20, Phillips, the Netherlands). Patients were pretreated with aspirin 100 mg/day and clopidogrel 75 mg/day for at least 5 days prior to the procedure, until an adequate response to both aspirin (AA inhibition >70%) and clopidogrel (ADP inhibition >30%) was detected by thromboelastography (TEG). Heparin was given as an intravenous bolus dose of 50 U/kg before the procedure, and a continuous flushing sodium solution (2.5 U/ml) was administered in arterial lines during the procedure(activated clotting time [ACT] maintained around 150-250 s). After general anesthesia and femoral artery cannulation, a 6 F Envoy guiding catheter (Cordis Neurovascular, USA) was placed into the artery proximal to the target lesion. Over a 200-cm Synchro-14 guidewire (Stryker Neurovascular, USA), an Echelon 10 microcatheter (ev3 Neurovascular, USA) was navigated to the distal part of the stenosis. The Synchro guidewire was then retrieved, and through the microcatheter, a 300-cm Transcend exchange wire (Boston Scientific, USA) was advanced into the artery distal to the stenosis. Pre-stent angioplasty was performed with the Gateway balloon (Stryker Neurovascular, USA), balloon sizes were selected to be similar to at least 80% of the diameter of the vessel either proximally or distally to the stenosis, balloons were slowly inflated (1 atm per 10–15 s) up to the nominal pressure. For consecutive stenting, an XT-27 microcatheter (Stryker Neurovascular, USA) was advanced bypass the stenosis over the exchange wire, the Neuroform EZ stent (Stryker Neurovascular, USA) was advanced through the XT-27 microcatheter and positioned until the stenosis was centered between the ends of the stent, and then deployed. After the procedure, intravenous heparin was maintained for the first 24 h, followed by aspirin 100 mg/day and clopidogrel 75 mg/day for at least 6 months, then one (with more optimal platelet inhibition) of the dual antiplatelet agents was administered daily thereafter.

### Follow-up

Follow-up information on clinical and angiographic outcomes was reviewed and collected by a trained neurointerventionist (BL). Clinical follow-up information was obtained from hospital records, in-person visit or telephone interview at 1, 3, 6, and 12 months, and yearly thereafter. Angiographic follow-up was scheduled at ~6 and 12 months. DSA was routinely used to access the vascular outcomes unless patients refused invasive assessment, in which CTA was used.

### Outcomes assessment

The preoperative and postoperative residual stenosis rate was calculated according to the WASID method (14). The pre- and postoperative neurological status was assessed using mRS and Institutes of Health Stroke Scale (NIHSS). The following data were collected consecutively:

- Technical feasibility: Defined as accurate delivery and deployment of the stent at the site of the target lesion, and improvement of the stenosis to less than 30%.
- Early complications: Defined as the 30-day rate of any procedure-related vascular event (TIA or stroke attributable to the territory of the target artery). A TIA was defined as any ischemic event resulting in a transient neurological deficit but resolved within 24 h. A stroke was defined as any hemorrhagic or ischemic event resulting in a new neurological deficit (scaled by the NIHSS and mRS score) lasting −24 h.
- Late complications: Defined as combined procedure-related permanent neurologic morbidity (scaled by the mRS score) and mortality rate beyond 30 days after PTAS.
- In-stent restenosis (ISR): Defined as luminal diameter stenosis over 50% for at least 6 months after the procedure.

## Results

### Patients

Between November 2016 and January 2018, 45 consecutive patients underwent Neuroform EZ stenting for symptomatic ICAS, a total of 46 intracranial atherosclerotic lesions were included. Intracranial stenting was performed only when (1) dual antiplatelet medication (aspirin and clopidogrel for at least 3 months) failed to prevent further ischemic events, (2) at least 2 weeks after the new-onset stroke and (3) non-atherosclerotic etiology were excluded. Our Preliminary indications of the Neuroform EZ stent use included (1) lesions involved perforator-bearing segments, (2) lesions in small vessels, (3) lesions with tortuous access vessel, which rendered potential access failure of other stents, and (4) lesions at bifurcations, e.g., top of the basilar artery, distal M1 segments.

Among all the lesions, 2.2% (*n* = 1) located in the distal internal carotid artery (ICA) in, 52.2% (*n* = 24) in the middle cerebral artery (MCA), 10.9% (*n* = 5) in the distal vertebral artery (VA) and 34.8% (*n* = 16) in the basilar artery (BA); Mori type A lesions were 23.9% (*n* = 11), type B were 60.9% (*n* = 28) and type C were 15.2% (*n* = 7); perforator branches involved in 58.7% (*n* = 27) of the lesions. The lesion-related presentations prior to procedure were classified as (1) hypoperfusion without stroke in 35.6% (*n* = 16), (2) hypoperfusion stroke 51.1% (*n* = 23), and (3) hypoperfusion with perforator strokes in 55.6% (*n* = 25). The baseline characteristics of the patients are shown in Table 1.

**Table 1:** Baseline characteristics of the patients.

**Baseline characteristic**	**Result**
Number of patients	45
Age	65 ± 10.8
**GENDER**	
Male	29 (64.4%)
Female	16 (35.6%)
**LESION LOCATION**	
Distal ICA	1 (2.2%)
MCA	24 (52.2%)
Distal VA	5 (10.9%)
BA	16 (34.8%)
**LESION MORPHOLOGY**	
Mori Type A	11 (23.9%)
Mori Type B	28 (60.9%)
Mori Type C	7 (15.2%)
Perforators involvement	27 (58.7%)
No perforators involvement	19 (41.3%)
**PREPROCEDURAL PRESENTATIONS**	
Hypoperfusion without stroke	16 (35.6%)
Hypoperfusion stroke	23 (51.1%)
Hypoperfusion with perforator strokes	25 (55.6%)

### Technical feasibility and vascular outcome

**Figure 1:**
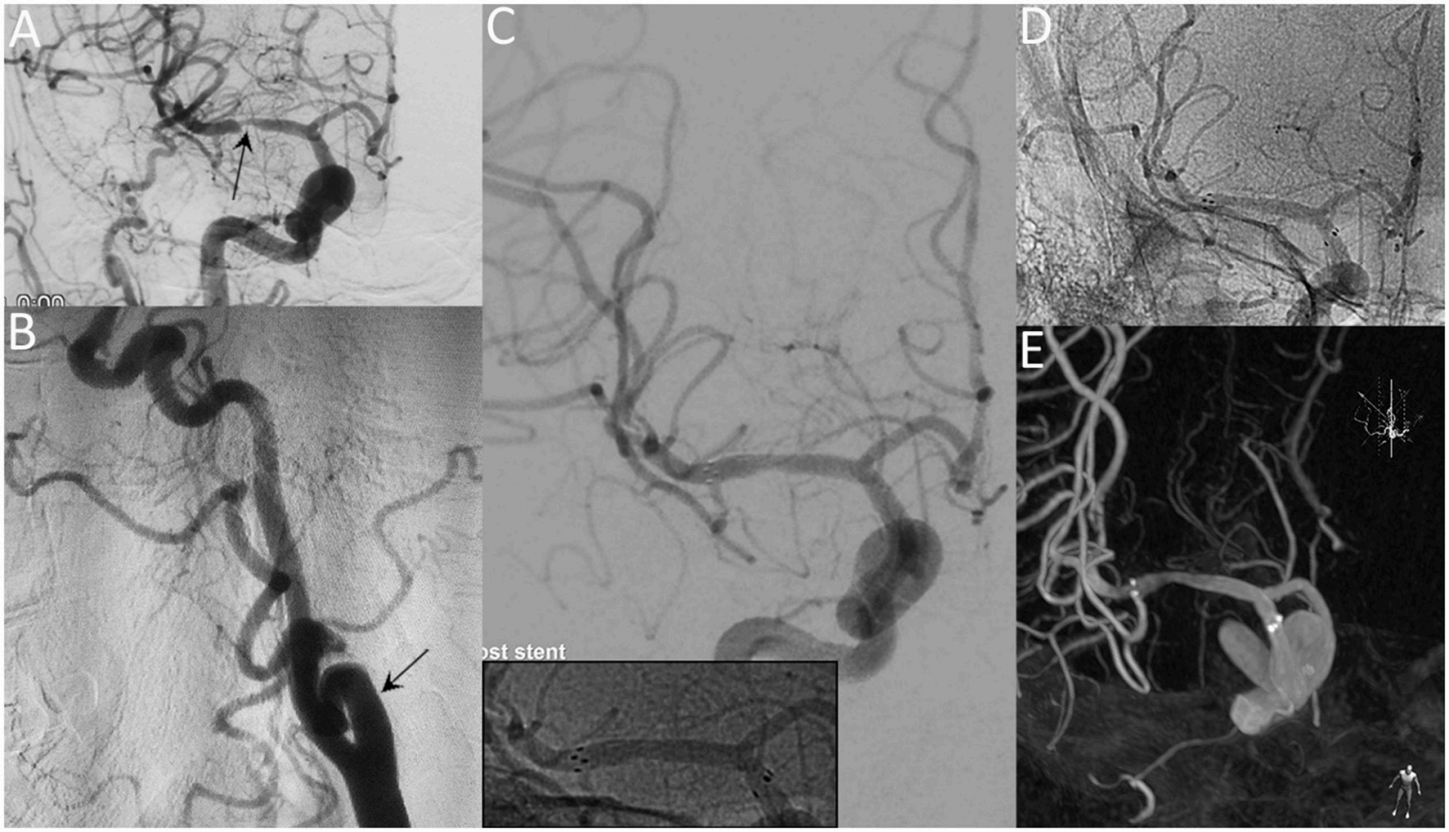
A 65-year-old female with a significant symptomatic stenosis of the right MCA. Preprocedural DSA images demonstrate high-grade stenosis (arrow) **(A)** and the tortuous right ICA (arrow) **(B)**. Postprocedural DSA images **(C)** reveal a residual stenosis of 10% (Insert: radiopaque markers of the stent). Unsubtracted angiogram **(D)** and XperCT **(E)** reveal patent stent with good apposition at the 8-month follow-up.

**Figure 2:**
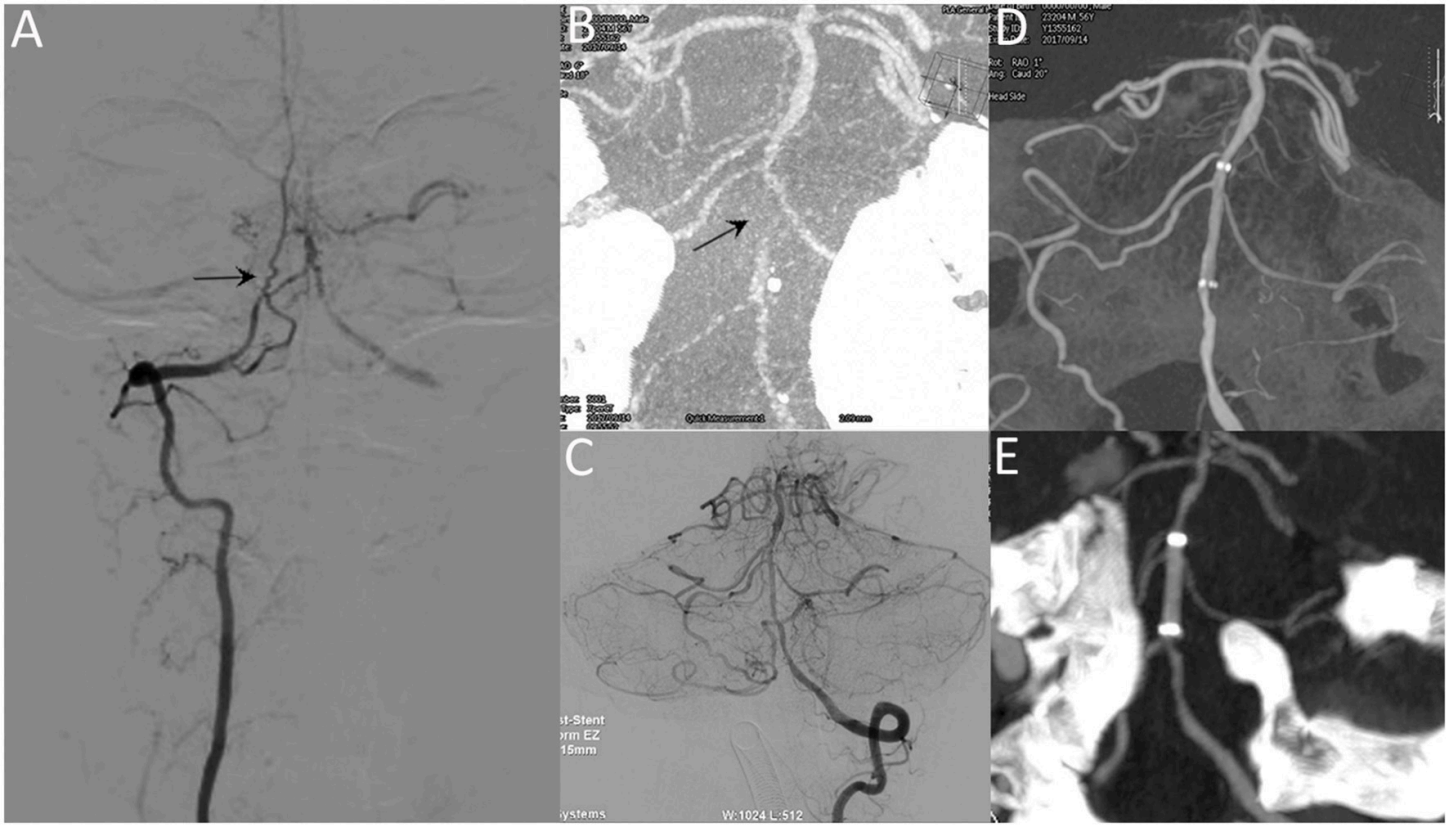
A 53-year-old male with repeated episodes of posterior circulation transient ischemic attacks. Preprocedural DSA image **(A)** and VasoCT **(B)** reveal near-occlusion of the basilar artery (arrow). Postprocedural DSA images **(C)** reveal patency of the basilar artery. Postprocedural XperCT **(D)** demonstrates good wall apposition of the stent. Follow-up CTA image **(E)** reveals the patent stent with good apposition 6 months after the procedure.

**Figure 3:**
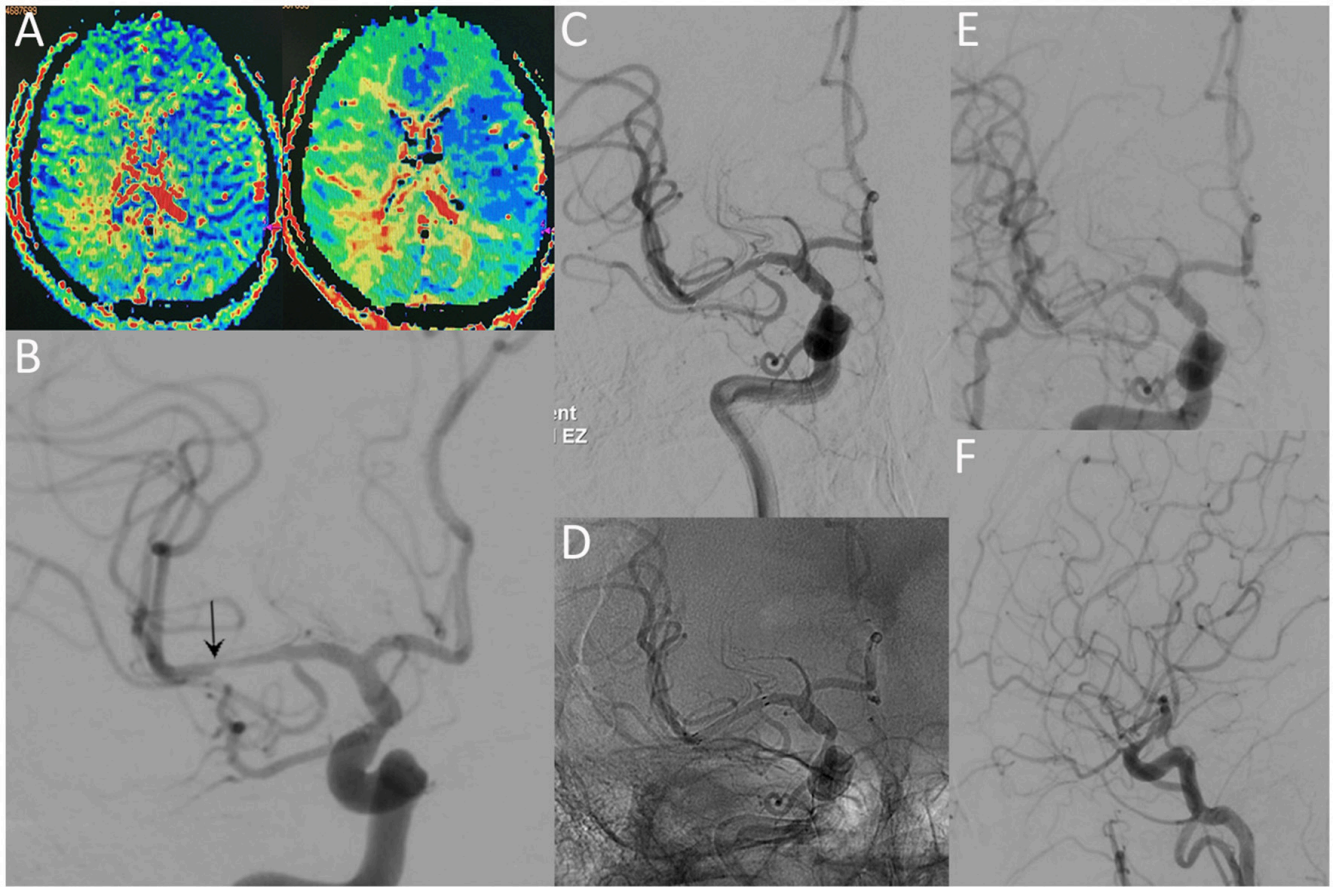
A 36-year-old male with a significant stenosis in the right MCA. CT perfusion images **(A)** reveal a prolongation of MTT and TTP before the procedure. Preprocedural DSA image **(B)** demonstrates high-grade stenosis close to the MCA bifurcation (arrow). Postprocedural DSA images **(C,D)** reveal patency of the MCA without residual stenosis. The 12-month follow-up DSA images **(E,F)** reveal no ISR in the region of the originally stented lesion.

The technical success rate was 100%. After treatment, the degree of stenosis was reduced to less than 30% (mean 23.7 ± 18.1%). Catheter angiography or computed tomography angiography (CTA) follow-up were obtained in 33 patients (73.3%). No recurrent stenosis was noted in all these patients with a mean follow-up period of 7.3 month. In total, 12 patients (26.7%) refused to undergo repeated DSA or CTA examination during the follow-up period. Of these, the preoperative and postoperative stenosis rate was (80.7 ± 7.3%) and (20.7 ± 13.7%), respectively. No deterioration of neurological function was noted in the clinical follow-up (mean 8.8 month) records of all these patients. Between different subgroups (Anterior circulation vs. Posterior circulation, Perforator-bearing vs. Non-perforator-bearing), no significant different was found in the outcomes. For the primary outcomes of different lesion types, differences between the subgroups were not statistically significant. The clinical variables of the patients in the different subgroups are shown in Table 2. Examples of Neuroform EZ stenting for ICAS are provided in Figures 1–3.

**Table 2:** The clinical variables of the patients.

	**All lesions**	**Anterior circulation**	**Posterior circulation**	**Perforator-bearing**	**Non-perforator-bearing**
No. of lesions	46	25	21	26	20
Preoperative stenosis rate (%)	86.5 ± 8.7	84.1 ± 7.1	89.6 ± 9.6^a^	82.7 ± 7.5	92.1 ± 7.1^b^
Postoperative stenosis rate (%)	23.7 ± 18.1	22.1 ± 12.8	24.6 ± 19.4^a^	20.4 ± 14.5	27.5 ± 15.7^b^
Average follow-up time (months)	8.6	8.9	8.2	8.5	8.8
Procedure-related complication at 30 days *n* (%)	1(2.2)	1(4)	0	1(3.8)	0
In-stent restenosis rate	0	0	0	0	0

a *compared with the anterior circulation group (p > 0.05)*,

b *compared with the perforator-bearing group (p > 0.05)*.

### Complications

**Figure 4:**
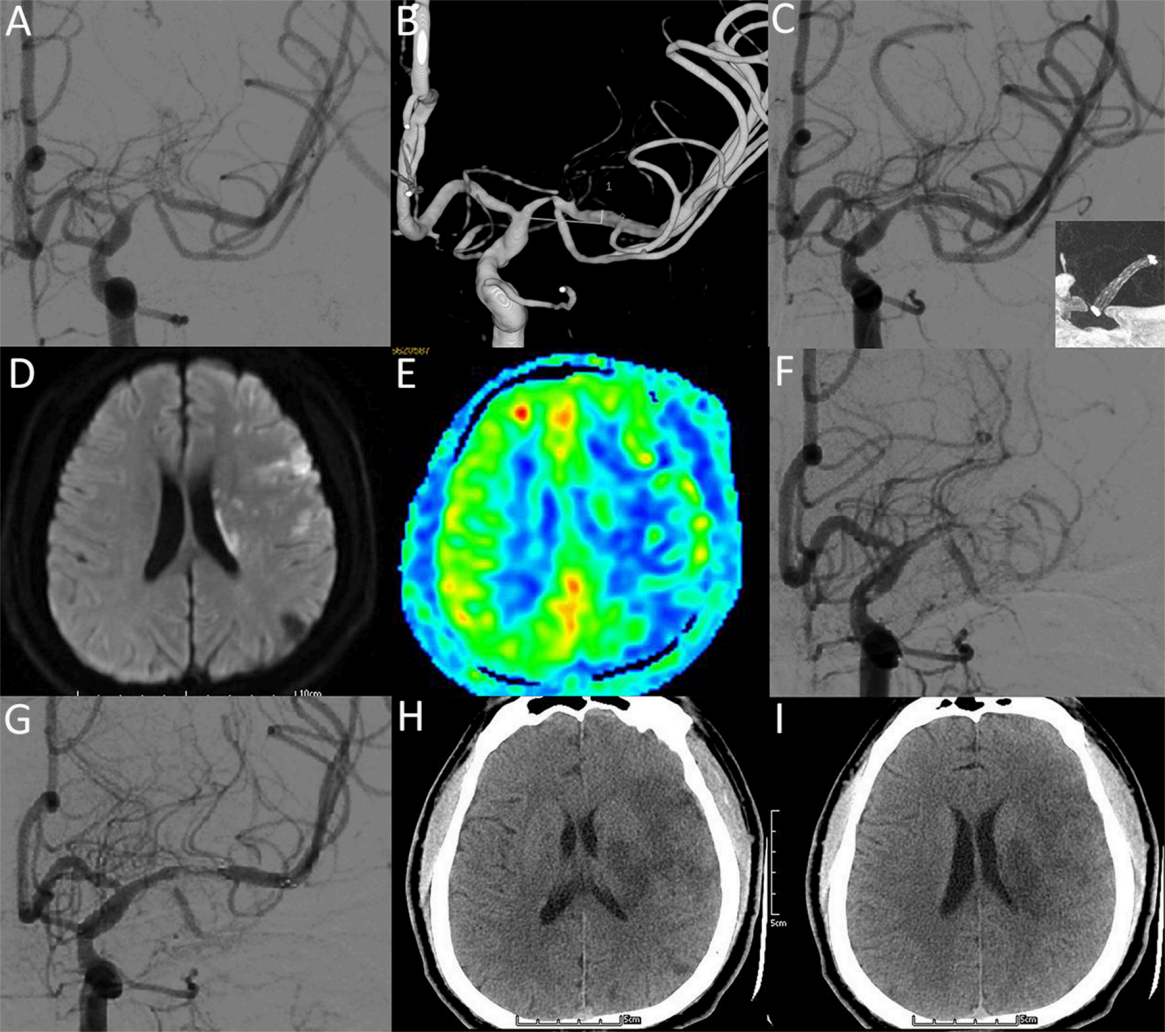
A 28-year-old male with a significant symptomatic stenosis of the left M1 segment. **(A,B)** Preprocedural DSA image demonstrates high-grade stenosis. **(C)** Postprocedural DSA image demonstrates a 20% residual stenosis (NIHSS: 0). Insert: VasoCT showing patent stent lumen with good stent vessel apposition. **(D-F)** Two days after stenting, DWI **(D)** and ASL **(E)** images reveal new infarctions and hypoperfusion in the territories of the left MCA (NIHSS: 14). DSA image **(F)** demonstrates complete occlusion of the left MCA. After the intra-arterial administration of IIb/IIIa inhibitor and solitaire stent deployment, DSA image **(G)** demonstrates recanalized flow of MCA (NIHSS: 14). Five days after recanalization, CT images **(H,I)** reveal hypointense lesions within the territories of the left MCA (NIHSS: 10).

- Early complications: The combined procedure related any vascular event rate within 30 days was 2.2% (*n* = 1). The patient was a 28-year-old male, he presented with recurrent episodes of right-sided hemianesthesia and aphasia over the prior year. He had a medical history of hypertension (first diagnosed 3 years ago), smoking (15 years, 10 cigarettes per day), alcohol consumption (10 years, 500 ml of white liquor per day) and a right anterior cerebral artery stroke (3 years previously, with no obvious sequelae, mRS = 0). A preprocedural angiogram revealed high-grade stenosis of the left M1 segment. The procedure was performed uneventfully (Gateway 15/9 mm for pre-stent angioplasty, Neuroform EZ 3/20 mm for stenting), with a 20% residual stenosis of the artery. When the patient regained consciousness after general anesthesia, no neurological deficit was detected. Forty-eight hours after the procedure he began to develop numbness and weakness in his right leg and arm (4^+^/5 power, NIHSS = 2). An urgent CT scan revealed no hemorrhagic changes, and intravenous IIb/IIIa inhibitor (tirofiban) was administered. The patient deteriorated within 6 h after the symptom onset, MR images reveal new infarctions and hypoperfusion in the territories of the left MCA. He had 1/5 power in the right upper limb and 2/5 in the right lower limb with slurred speech (NIHSS = 14) before a second procedure. During the second procedure, DSA image demonstrates complete occlusion of the left MCA. After the intra-arterial administration of IIb/IIIa inhibitor and solitaire stent (6/20 mm) deployment, the occluded MCA was gradually recanalized (TICI 3). No significant improvement of neurological functions was achieved after the procedure. The patient was discharged 50 days later, maintained right-sided plegia and slurred speech (NIHSS = 10, mRS = 5) (Figure 4).
- Late complications: No procedure related permanent neurologic morbidity and mortality rate beyond 30 days was noted during 8.6 months of mean follow-up.

## Discussion

### Neuroform EZ: a promising optional device for ICAS stenting

As a self-expanding intracranial stent, the Neuroform stent was initially developed to assist the coiling of wide-necked intracranial aneurysms (8). Subsequently, the Wingspan stent was developed for the endovascular treatment of ICAS as a design variant with an optimized delivery system and radial force (9). However, the Gateway-Wingspan system failed in the SAMMPRIS trial for creating too many complications (4). Yet, despite advances in medical therapy, patients with recalcitrant ICAS may continue to experience TIAs and strokes, especially for the Asian population (10). It is these patients for whom the endovascular therapy was mandatory, even, recent examination in large trials showed no benefit of this intervention. Regarding various salient features of ICAS lesions, a lesion-specific design of procedures with dedicated devices might help to resolve this issue, instead of the exclusive option for devices approved by the Food and Drug Administration (FDA). As an alternative, the Neuroform stent had been used for ICAS in several case series (11–13). Thus, for the relatively small sample size (3 trials, 14 patients totally), beyond the scope of the indicated uses outlined in the device manual, the experience of the Neuroform EZ stenting for ICAS was still limited. In the present study, our results revealed that selected ICAS stenting with Neuroform EZ stent was safe and efficacious. Moreover, focused analysis of lesions of this series would help neurointerventionists in selecting the most appropriate device for ICAS stenting on the basis of individualized decision making.

### Neuroform EZ for ICAS stenting:relationship between device physical properties and procedural outcome

**Figure 5:**
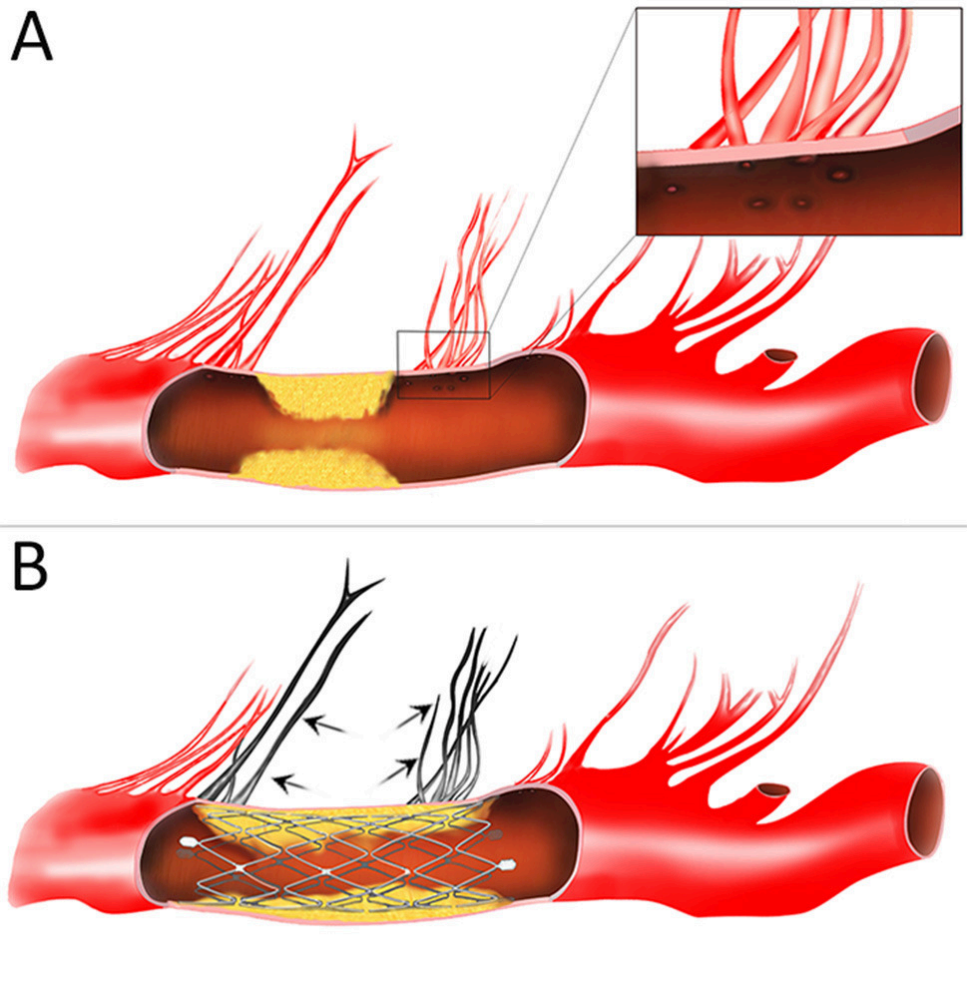
Schematic diagram of the “snow-plowing” effect. **(A)** An intracranial atherosclerotic arterial stenosis with perforator vessels. Inset: the magnified view of perforator ostia. **(B)** After stent deployment, forceful displacement of the plaque causes occlusion of the perforator vessels (arrow).

In the prematurely halted SAMMPRIS trial, poor outcomes are largely attributable to ischemic stroke secondary to perforator branch occlusion (4, 15). The mechanism of perforator stroke has mainly been demonstrated by plaque shift after deployment of a stent with relative higher radial force (16). Although higher radial force may result in larger lumen for achieving improved flow, the atheromatous debris of the plague entrapped between expanding stent struts and the arterial wall might be forced into perforator ostia (Figure 5), which was termed “snowplowing” effect and may pose a major risk of perforator occlusion related to stenting. Hence, device-related complications should be carefully taken into consideration in the stents selection for ICAS. In an *in vitro* examination, while expanding the vessel diameter at about 85% of the labeled diameter, the Wingspan stent produces nearly a 0.5-fold increase in chronic radial strength as compared with Neuroform (17). Theoretically, given the physical properties, a stent with appropriate radial force might help to solve the “snowplowing” problem. In the present series, as we expected, with the utilization of Neuroform EZ stent, our results revealed that the periprocedural complication rates reduced to 2.2% (1), especially for a 7.2% drop of perforator event compare with the SAMMPRIS subgroup [15 [7.2%] of 21 [10.1%] for ischemic complications] (15). The rationale behind the present results might be that the Neuroform EZ stent was more flexible than Wingspan, exert reduced outward radial force. In another post-SAMMPRIS prospective trial for the individualized treatment of ICAS, with the exclusion criteria of perforator territory strokes before the procedure, positive results were also yielded in a subgroup with device selection limited to Wingspan (18). Thus, in our study, patients presented with perforator territory strokes were not excluded (55.6%), the outcome was even better than previous studies. This suggested that Neuroform EZ stenting may be beneficial in the selected subset of ICASs with a higher risk of procedure-related perforator infarct.

Another major influence on the outcome of ICAS stenting trials was the in-stent restenosis (ISR). As a device-related trigger of ISR, intimal hyperplasia may be stimulated by the outward radial force of the stent. The Wingspan stent is a self-expandable stent (SES) with a relatively high radial opening force. In contrast, the Neuroform EZ stent is a SES with reduced radial force compared to the Wingspan stent (19, 20). In this study, the angiographically demonstrated pre-procedural stenosis rate was 86.5 ± 8.7%, imaging follow-up (mean 7.3 months) revealed no ISR occurred, which was lower than published trials with stents of higher radial force (21, 22). In another group of patients treated with a drug-eluting coronary stent (DES), a substantially lower ISR of 3.8% could be achieved with an acceptable procedural complication rate of 0.9%. However, 7% of the procedures failed due to the high rigidity of the stent system (23). Under the hypothesis that more flexible stents will be less likely to result in ISR than one with high radial force, the Enterprise stent or the Solitaire stent also have been used in several series. Yet, compared to our results, the rate of ISR in these studies (24.7% for Enterprise, 11.4% for Solitaire) was not significantly lower (20, 24). Mechanically, appropriate rigidity should be taken into account when the more flexible device was adopted in ICAS stenting, so as to exert adequate radial force to resist the elastic recoil of the target vessel while promoting the ability to navigate in tortuous vessels. Certainly, there is no stent that is superior in all clinical and technical requirements. Therefore, clinical advantages of ICAS stenting should be based on the focused analysis of location and morphology of the ICAS lesions as well as device physical properties.

### Neuroform EZ for ICAS stenting:preliminary lesion-specific recommendations and modified techniques

The features of ICAS lesions is likely diversiform in nature. Different types of symptomatic intracranial stenosis may respond differently to interventional strategy. Even for skilled neurointerventionists, in case of ICAS stenting, positive outcomes would depend not only on patient selection, but also the procedural techniques with given specific devices. Based on our experience with the use of the Neuroform EZ stents for ICAS, the lesion-specific recommendations and modified techniques are preliminarily summarized as follows:

Lesion-specific recommendations: (1) lesions with tortuous access vessel (Figure 1), (2) lesions involving small vessels (Figure 2), (3) lesions close to or across a bifurcation (Figure 3).

**Figure 6:**
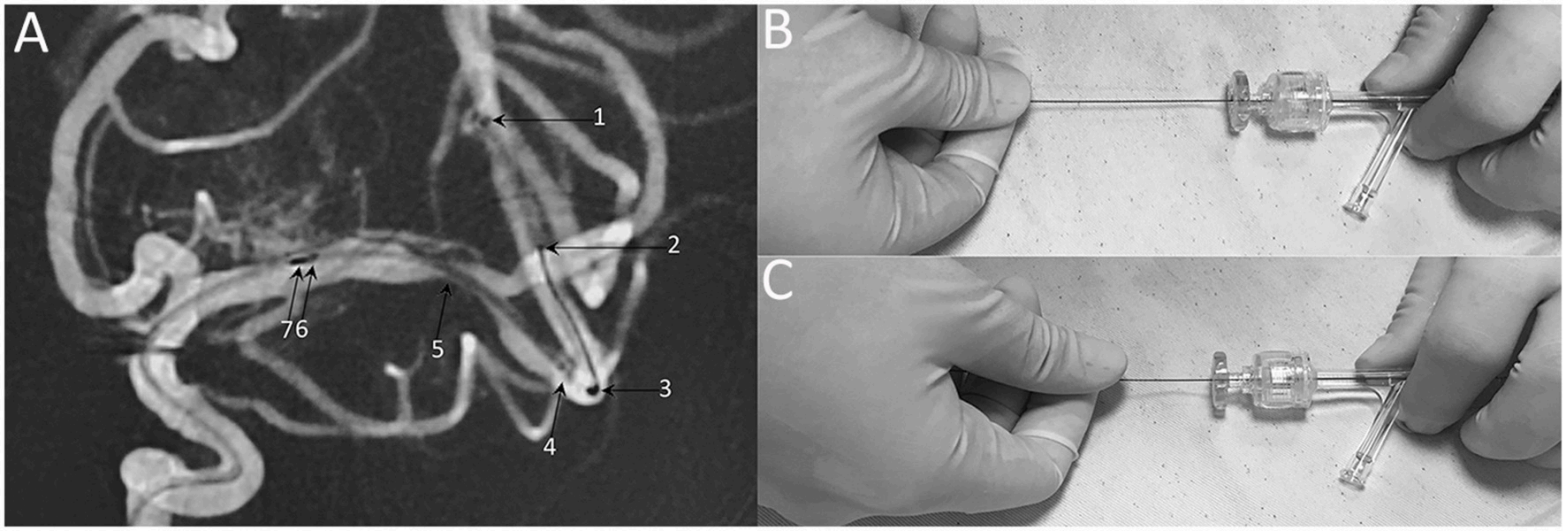
Technical note of Neuroform EZ stent deployment. **(A)** Representative DSA image of stent deploying, demonstrating the radiopaque markers of 1: XT-27 microcatheter tip, 2: distal tip of the delivery wire, 3: distal bumper, 4: distal stent markers, 5: stenosis 6: stent proximal markers, 7: proximal bumper. **(B)** When the stent was ready to be deployed, holding the stent delivery wire at a sufficient distance (about 8-10 cm) from the RHV. **(C)** Insufficient distance may cause unnecessary hand movement and poor stent apposition.

Modified techniques: (1) balloon and stent size selection: balloon sizes were selected to be similar to at least 80% of the diameter of the vessel either proximally or distally to the stenosis; the stent diameter was sized to exceed the diameter of the proposed artery by 0.5–1.0 mm, the stent length was selected to exceed the length of the lesion by at least 3 mm on both sides; (2) microcatheter position: the tip of XT-27 microcatheter should be distal enough to the lesion to preserve sufficient length of landing zone to accommodate the distal tip (19 mm) of the stent delivery wire (Figure 6A); (3) stent deployment: when the stent was ready to be deployed, holding the stent delivery wire at a sufficient distance (about 8–10 cm) from the RHV to avoid unnecessary hand movement during stent deployment (Figures 6B,C).

## Limitations of this study

First, this retrospective study was more prone to a certain bias compared with the prospective randomized trial. Second, our study was conducted at a single institution, and the population-specific experiences may not be globally generalized. Certainly, multicenter studies with larger sample size and long-term follow-up period are needed to confirm the clinical and angiographic results of this study.

## Conclusion

The off-label use of Neuroform EZ stent might lower procedural complications in stenting for many medically refractory ICASs. Our initial experience provides feasibility and safety data to guide future alternative procedures with Neuroform EZ in ICAS stenting. Due to the respective nature of this study, longer-term follow-up and further randomized trials are still mandatory to determine the durability and viability of our promising results.

## Ethics statement

This study was performed with approval from the institutional ethics committee of Chinese PLA General Hospital (NO:S2018-060-01). All patients or their authorized representative were explicitly informed and gave written informed consent to the off-label use of the Neuroform EZ stent.

## Author contributions

ZD and JM performed the analysis and wrote the paper. SY and CT contributed to data analysis. JW, ZD, XC, and XL performed the interventions and were responsible for patients care and management. RM, RZ, and BL contributed to acquisition of data. JW contributed to the study design and critical revision of the manuscript.

### Conflict of interest statement

The authors declare that the research was conducted in the absence of any commercial or financial relationships that could be construed as a potential conflict of interest.

## References

[1] Holmstedt CA Turan TN Chimowitz MI. Atherosclerotic intracranial arterial stenosis: risk factors, diagnosis, and treatment. Lancet Neurol. (2013) 12:1106–14. 10.1016/S1474-4422(13)70195-9 24135208 PMC4005874

[2] Fiorella D Levy EI Turk AS Albuquerque FC Niemann DB Aagaard-Kienitz B. US multicenter experience with the Wingspan stent system for the treatment of intracranial atheromatous disease: periprocedural results. Stroke (2007) 38:881–7. 10.1161/01.STR.0000257963.65728.e8 17290030

[3] Fiorella D Turk AS Levy EI Pride GL Woo HH Albuquerque FC. US wingspan registry: 12-month follow-up results. Stroke (2011) 42:1976–81. 10.1161/STROKEAHA.111.613877 21636812

[4] Chimowitz MI Lynn MJ Derdeyn CP Turan TN Fiorella D Lane BF. Stenting versus aggressive medical therapy for intracranial arterial stenosis. N Engl J Med. (2011) 365:993–1003. 10.1056/NEJMoa1105335 21899409 PMC3552515

[5] Zaidat OO Fitzsimmons BF Woodward BK Wang Z Killer-Oberpfalzer M Wakhloo A. Effect of a balloon-expandable intracranial stent vs medical therapy on risk of stroke in patients with symptomatic intracranial stenosis: the VISSIT randomized clinical trial. JAMA (2015) 313:1240–8. 10.1001/jama.2015.1693 25803346

[6] Turan TN Cotsonis G Lynn MJ Wooley RH Swanson S Williams JE. Intracranial stenosis: impact of randomized trials on treatment preferences of US neurologists and neurointerventionists. Cerebrovasc Dis. (2014) 37:203–11. 10.1159/000358120 24557055 PMC3991561

[7] Sangha RS Naidech AM Corado C Ansari SA Prabhakaran S. Challenges in the medical management of symptomatic intracranial stenosis in an Urban setting. Stroke (2017) 48:2158–63. 10.1161/STROKEAHA.116.016254 28679857 PMC5558843

[8] Henkes H Bose A Felber S Miloslavski E Berg-Dammer E Kühne D. *Endovascular coil* occlusion of intracranial aneurysms assisted by a novel self-expandable nitinol microstent (neuroform). Interv Neuroradiol. (2002) 8:107–19. 10.1177/159101990200800202 20594519 PMC3576604

[9] Henkes H Miloslavski E Lowens S Reinartz J Liebig T Kühne D. Treatment of intracranial atherosclerotic stenoses with balloon dilatation and self-expanding stent deployment (WingSpan). Neuroradiology (2005) 47:222–8. 10.1007/s00234-005-1351-2 15912418

[10] Mehndiratta MM Khan M Mehndiratta P Wasay M. Stroke in Asia: geographical variations and temporal trends. J Neurol Neurosurg Psychiatry (2014) 85:1308–12. 10.1136/jnnp-2013-306992 24769474

[11] Turk AS Ahmed A Niemann DB Aagaard-Kienitz B Brooks N Levine RL. Utilization of self-expanding stents in the treatment of intracranial atherosclerotic disease in the distal small cerebral vessels. Neuroradiology (2007) 49:659–63. 10.1007/s00234-007-0229-x 17387464

[12] Rohde S Seckinger J Hähnel S Ringleb PA Bendszus M Hartmann M. Stent design lowers angiographic but not clinical adverse events in stenting of symptomatic intracranial stenosis - results of a single center study with 100 consecutive patients. Int J Stroke (2013) 8:87–94. 10.1111/j.1747-4949.2011.00715.x 22296983

[13] Hähnel S Ringleb P Hartmann M. Treatment of intracranial stenoses using the neuroform stent system: initial experience in five cases. Neuroradiology (2006) 48:479–85. 10.1007/s00234-006-0081-4 16721557

[14] Samuels OB Joseph GJ Lynn MJ Smith HA Chimowitz MI. A standardized method for measuring intracranial arterial stenosis. AJNR Am J Neuroradiol. (2000) 21:643–6. 10782772 PMC7976653

[15] Fiorella D Derdeyn CP Lynn MJ Barnwell SL Hoh BL Levy EI. Detailed analysis of periprocedural strokes in patients undergoing intracranial stenting in Stenting and Aggressive Medical Management for Preventing Recurrent Stroke in Intracranial Stenosis (SAMMPRIS). Stroke (2012) 43:2682–8. 10.1161/STROKEAHA.112.661173 22984008 PMC3509932

[16] Fujimoto M Shobayashi Y Takemoto K Tateshima S Viñuela F. Structural analysis for wingspan stent in a perforator model. Interv Neuroradiol. (2013) 19:271–75. 10.1177/159101991301900302 24070074 PMC3806000

[17] Krischek Ö Miloslavski E Fischer S Shrivastava S Henkes H. A comparison of functional and physical properties of self-expanding intracranial stents [Neuroform3, Wingspan, Solitaire, Leo(+), Enterprise]. Minim Invasive Neurosurg. (2011) 54:21–8. 10.1055/s-0031-1271681 21506064

[18] Miao Z Song L Liebeskind DS Liu L Ma N Wang Y. Outcomes of tailored angioplasty and/or stenting for symptomatic intracranial atherosclerosis: a prospective cohort study after SAMMPRIS. J Neurointerv Surg. (2015) 7:331–5. 10.1136/neurintsurg-2014-011109 24759694 PMC4207720

[19] Kim M Levy EI Meng H Hopkins LN. Quantification of hemodynamic changes induced by virtual placement of multiple stents across a wide-necked basilar trunk aneurysm. Neurosurgery (2007) 61:1305–12. 10.1227/01.neu.0000306110.55174.30 18162911 PMC2756037

[20] Vajda Z Schmid E Güthe T Klötzsch C Lindner A Niehaus L. The modified Bose method for the endovascular treatment of intracranial atherosclerotic arterial stenoses using the Enterprise stent. Neurosurgery (2012) 70:91–101. 10.1227/NEU.0b013e31822dff0f 21778921

[21] Albuquerque FC Levy EI Turk AS Niemann DB Aagaard-Kienitz B Pride GL Jr. Angiographic patterns of Wingspan in-stent restenosis. Neurosurgery (2008) 63:23–7. 10.1227/01.NEU.0000335067.53190.A2 18728565

[22] Zhu SG Zhang RL Liu WH Yin Q Zhou ZM Zhu WS. Predictive factors for in-stent restenosis after balloon-mounted stent placement for symptomatic intracranial atherosclerosis. Eur J Vasc Endovasc Surg. (2010) 40:499–506. 10.1016/j.ejvs.2010.05.007 20554461

[23] Vajda Z Aguilar M Göhringer T Horváth-Rizea D Bäzner H Henkes H. Treatment of intracranial atherosclerotic disease with a balloon-expandable paclitaxel eluting stent procedural safety, efficacy and mid-term patency. Clin Neuroradiol. (2012) 22:227–33. 10.1007/s00062-011-0125-y 22252289 PMC3432207

[24] Duan G Feng Z Zhang L Zhang P Chen L Hong B. Solitaire stents for the treatment of complex symptomatic intracranial stenosis after antithrombotic failure: safety and efficacy evaluation. J Neurointerv Surg. (2016) 8:680–4. 10.1136/neurintsurg-2015-011734 26041096

